# The impacts of training on change deafness and build-up in a flicker task

**DOI:** 10.1371/journal.pone.0276157

**Published:** 2022-11-17

**Authors:** Natalie Ball, Matthew Wisniewski, Brian Simpson, Eduardo Mercado

**Affiliations:** 1 U.S. Air Force Research Laboratory, Wright-Patterson Air Force Base, Dayton, OH, United States of America; 2 Department of Psychological Sciences, Kansas State University, Manhattan, KS, United States of America; 3 Department of Psychology, University at Buffalo, Buffalo, NY, United States of America; University Medical Center Goettingen, GERMANY

## Abstract

Performance on auditory change detection tasks can be improved by training. We examined the stimulus specificity of these training effects in behavior and ERPs. A flicker change detection task was employed in which spatialized auditory scenes were alternated until a "change" or "same" response was made. For half of the trials, scenes were identical. The other half contained changes in the spatial locations of objects from scene to scene. On Day 1, participants were either trained on this auditory change detection task (trained group), or trained on a non-auditory change detection task (control group). On Day 2, all participants were tested on the flicker task while EEG was recorded. The trained group showed greater change detection accuracy than the control group. They were less biased to respond "same" and showed full generalization of learning from trained to novel auditory objects. ERPs for "change" compared to "same" trials showed more negative going P1, N1, and P2 amplitudes, as well as a larger P3b amplitude. The P3b amplitude also differed between the trained and control group, with larger amplitudes for the trained group. Analysis of ERPs to scenes viewed prior to a decision revealed build-up of a difference between "change" and "same" trials in N1 and P2. Results demonstrate that training has an impact early in the "same" versus "change" decision-making process, and that the flicker paradigm combined with the ERP method can be used to study the build-up of change detection in auditory scenes.

## 1. Introduction

We often believe our perception of the world to be vivid and detailed, but large changes can escape our detection. *Change deafness* is a phenomenon where individuals fail to detect changes to an auditory environment even when those changes are very apparent [1]. Change deafness occurs with environmental sounds [e.g. human, object, animal, musical sounds) [2–10], harmonic and disharmonic chords [11–13], and speech [14,15]. Change deafness can occur even when auditory scenes are sparse, with the number of sound sources (auditory objects) as low as four [16]. It has also been observed with different kinds of changes, such as object deletion [4,5], object addition [5,17], object replacement [6,10,16,18], and object location changes [4,8,10,19]. Generally, it is considered to be the auditory counterpart to the much more frequently studied *Change Blindness* phenomenon [20,21].

The study of change deafness is important for understanding the nature of perceptual errors, which can be problematic or even dangerous in real-world situations. Changes in sonic environments provide information about events as they unfold, such as the sound of a car horn during street crossing, the beeps of a cardiac monitor amongst the sounds of an emergency room, and the change in direction or distance of gunfire in battle. Understanding the perceptual and cognitive processes that underlie auditory change detection can inform the development of interfaces to enhance human performance, and procedures that are adapted to alleviate change deafness and other common perceptual errors.

### 1.1 Impacts of learning on change detection

One way to potentially ameliorate change deafness is through training. Experience and practice with auditory tasks has been shown to improve spatial acuity, discrimination, and signal detection [22–24]. Few studies, however, have looked at how training impacts detection of changes in scenes. Irsik [25] investigated whether change deafness can be reduced by training, and if consolidation continued to affect performance beyond the initial learning period. Participants were trained with or without feedback using rhythmic scenes made up of band-pass filtered noise. A “one-shot” task was used wherein two scenes were presented in succession with a “change”/“same” response following the presentation of both scenes [e.g. 26,27]. Participants were tested before, immediately after, and 12 hours following training. Results showed that training increased sensitivity to changes. Additionally, response bias was reduced by training with feedback; specifically, feedback-trained participants showed a more neutral strategy of response compared to those trained with no feedback, who were more inclined to respond “same.” In the visual domain, Gaspar et al. [28] and Rosen et al. [29] both found that improvements during training were specific to scenes experienced in training, with learning related to familiarity with the stimuli. Pre-exposure to visual objects has been shown to decrease ability to perceive changes that were *easily detected* without pre-exposure, but also increase ability to perceive changes that were *less easily detected* without pre-exposure These effects were revealed using a change blindness "flicker” task wherein scenes alternated several times, allowing several observations and presumably checking of multiple locations within scenes.

ERPs provide temporally-sensitive information on neural processes as they unfold and have yielded valuable information about learning effects outside the scope of change deafness work. For example, musicians have been shown to have larger N1-P2 responses to sound, likely related to learning history [30]. N1, P2, MMN, and P3 enhancement have all been reported after training on auditory tasks [31–35]. Orduña et al. [35] trained individuals with complex non-speech sounds and found that this training was accompanied by increases in the P2 component of the auditory ERP that accompanied improvements in sound discrimination. Though the sounds used in that study were not made up of scenes with multiple auditory objects, the three-alternative forced-choice task used to test performance did assess ability to discern changes from one interval to the next, making it a change detection task. Recently, Wisniewski et al. [22] found that listeners were better at detecting tones at a frequency they were trained with (either 861-Hz or 1058-Hz), and that P2 and P3 amplitudes were larger for trained than for untrained tones during a listener’s active engagement in detection (also, see [36]). However, even during passive exposure to these sounds, P2 amplitudes were larger for trained than for untrained tones. These results suggest that detection training can induce changes in relatively early perceptual processing, as well as have an impact on later processing that takes place when a listener is actively trying to detect sounds. No studies, however, have used ERPs to study the processes that might underlie the impacts of training on change deafness in auditory scenes. One goal of this study was to fill this gap in the literature.

### 1.2 ERP studies of change deafness

Auditory N1, P2, and P3 amplitudes have all been identified as correlates of auditory change detection. Gregg and Snyder [6] found larger N1 amplitudes for detected compared to undetected change trials, whereas P2 was larger for trials where a change was not detected (compared to no-change trials). This led the authors to conclude that N1 may indicate some physiological change detection mechanism, leading to conscious detection, whereas P2 does not (also see [37,38]). Ball et al. [10] found that N1-P2 complexes were largest for trials in which a change in the auditory scene was detected, but was misidentified. Considering this research together, it seems possible that N1 and P2 are associated with an “implicit” detection of change that does not necessarily equate to accurate change detection through later cognitive processing. Changes may be successfully encoded, but then fail to trigger these higher-order processes that relate to the successful detection of change or the type of change that occurred. Unlike the earlier ERP components associated with sensory processing, the later P3 component has been consistently found to be larger in amplitude for successful change detection [6,10,37,38]. Additionally, P3 has been associated with change detection with different types of change (e.g. spatial and identity changes), indicating that P3 is a reliable indicator of change detection, regardless of change type [10].

All of the aforementioned studies on ERPs and change deafness have employed the one-shot task paradigm. A second goal of this work was to conduct an initial examination of ERP correlates of change deafness in a flicker task. Little is known about how information accumulation affects the detection of change in auditory environments. P2 and P3 have been found to increase in amplitude as sequences of stimuli proceed, referred to as “evidence accumulation” over time as part of the decision-making process [39,40]. N1 has also been shown to increase in amplitude prior to the detection of a tone within a complex auditory scene [41]. Vierck and Kiesel [42] found that detection was improved with increasing repetitions in a change blindness flicker task, showing that flicker tasks are useful for studying evidence accumulation. Although no studies have directly examined ERP accumulation effects with an auditory flicker task, it is possible that as acoustic scenes alternate, ERP component amplitude increases might be seen as evidence is accumulated prior to a response. Such an effect would allow the characterization of the temporal dynamics of auditory change detection/deafness in a novel way. For instance, the flicker task could be used to study the build-up of change detection at early sensory and later processing stages.

### 1.3 The current study

Here, a flicker task was used in both training and testing. Environmental scenes were presented that entailed either changes in the spatial locations of objects (50% of trials) or no change in scenes at all. These scenes were alternated until a response was made. In training, participants heard the same four sounds in all scenes (trained group) or trained in a non-auditory visual task (control group). In testing, listeners heard scenes made up of either “trained” or “novel” sounds. "Trained" and "novel" sounds were arbitrary for the control group as no auditory training was given. It was hypothesized that the trained group would outperform the control group in testing, and that analysis of ERPs would indicate a difference between groups characterizing the processing stage (or stages) at which training had effects. In regard to the novel use of a flicker paradigm, we expected that ERPs would reveal differences between "change" and "same" trials replicating previous work with the one-shot paradigm. We also expected that analysis of ERPs up until a response would show a build-up of these differences between "change" and "same" trials.

## 2. Methods

### 2.1 Participants

Twenty young adults age 18–40 years old (*M* = 23.33, *SD* = 5.94) with normal hearing, confirmed by standard audiometric testing (<20 dB HL, 0.25–8 kHz), participated for compensation. All participants had some previous experience with psychoacoustic tasks. These studies were approved by the Wright Site Institutional Review Board. All participants gave written, informed consent. Participants were quasi-randomly assigned to ensure equal participant numbers in the training condition (age: *M* = 23.50 years, *SD* = 4.93) and control condition (age: *M* = 23.50 years, *SD* = 7.28).

### 2.2 Apparatus

Participants sat in a sound-attenuating booth during testing. Sounds were presented through insert earphones (ER-2; Etymotic Research, Elk Grove Village, IL, USA) at a level not

exceeding 80 dB Sound Pressure Level (SPL). Timing of stimuli was controlled using a

TDT System 3 real-time processor (RP2.1; Tucker-Davis Technologies, Alachua, FL). Experimental procedures were executed in MATLAB (Mathworks, Natick, MA).

### 2.3 Stimuli

The auditory objects were eight environmental sounds (see Table 1) selected from a commercially available compilation of sound effects [43]. These sounds were selected for their similarity in bandwidth and for their identifiability (based on prior behavioral testing, see [19]). Further, these sounds have been used previously in a study demonstrating N1, P2, and P3b correlates of change deafness [10].

**Table 1:** Sounds used in this experiment, along with percentages of sound recognition in a normal recognition task from Simpson et al. [19].

Sound	Recognition Accuracy	Sound	Recognition Accuracy
Teethbrushing	96%	Harp	99%
Dog bark	100%	Lawnmower	100%
Pig	99%	Telephone	100%
Firetruck	100%	Bees	99%

Sounds were filtered to a bandwidth of 0.2 kHz–14 kHz and normalized so that all sounds had the same overall RMS amplitude. Scenes comprised four objects and were presented for a duration of 500 ms (if original sound duration was not long enough to fill the 500ms, it was looped during presentation) with a 400 ms ISI of silence between scenes. Sounds were separated in virtual space on the listener’s horizontal plane at 20°, 5°, −5°, and −20° from center. Head-related transfer function (HRTF) measurements from KEMAR dummy head recordings were used to render these azimuths. These recordings were made through the in-ear microphones on KEMAR (GRAS 46A) in the Auditory Localization Facility at Wright-Patterson Air Force Base. The facility consists of an anechoic chamber, the walls, floor, and ceiling of which are covered with 1.1 m thick fiberglass wedges to reduce echoes. This chamber houses a seven-foot geodesic sphere, 4.3 m in diameter, with 277 BOSE 11 cm, full-range loudspeakers mounted on its surface. Broadband chirp stimuli were played from each location to the KEMAR dummy and HRTFs were formed from these recordings (further detail is described in [44]).

### 2.4 Procedure

**Fig 1:**
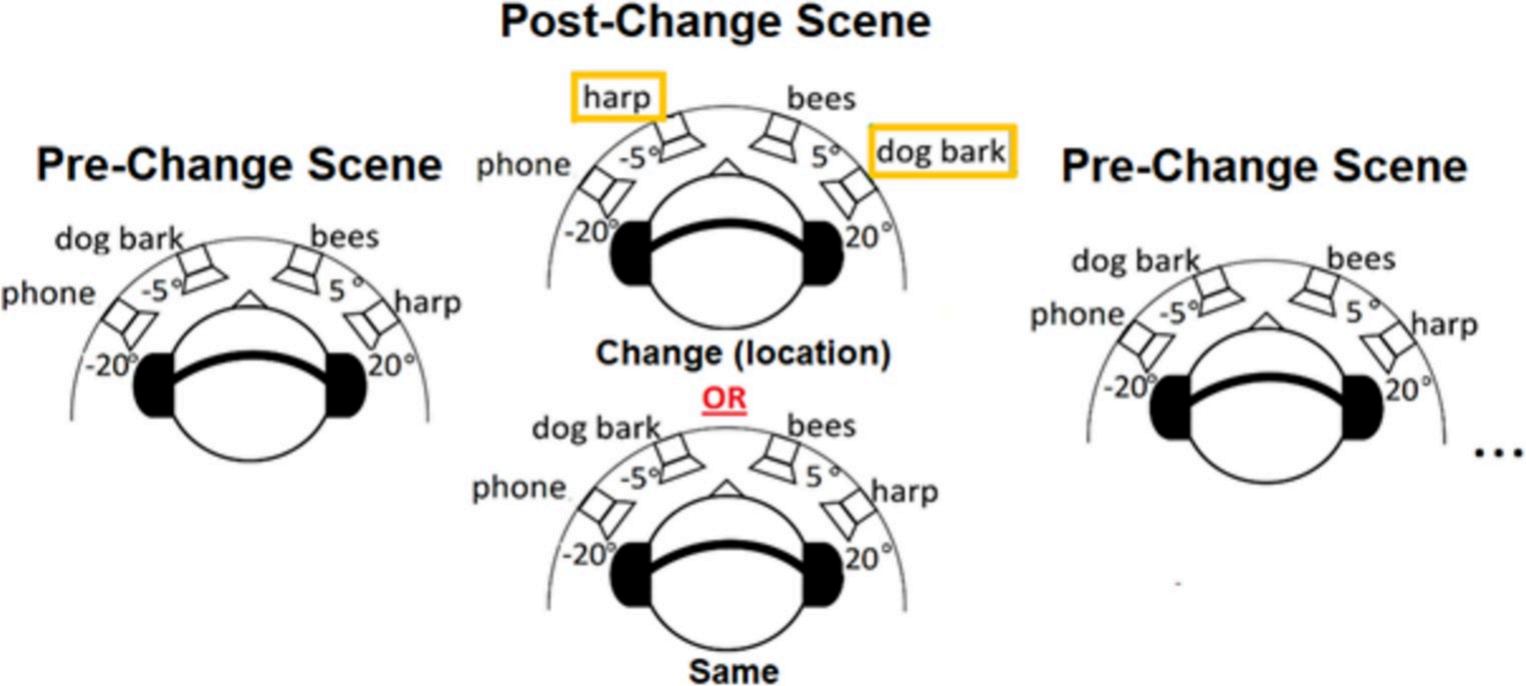
Examples of the two types of trials. The pre-change was presented first (left), then the post-change scene consisted of either a location change (switch of objects on the horizontal plane) or was the same as the pre-change scene. The pre- and post-change scene alternated until participant makes a response or eight cycles were presented.

Each participant had a set of four randomly-assigned “trained” and four “novel” sounds. Trained participants only heard their “trained” sounds during training, and then heard both “trained” and “novel” sounds (in separate trials scenes) during testing. Note that the random assignment of different sounds to "trained" and "novel" makes it difficult to assess any hypotheses related to the detectability of changes for different sounds, but that this was not a hypothesis of interest here. Sounds were selected to be matched on localizability from pilot testing to help mitigate such potential effects. Control participants engaged in a non-auditory change detection task during their “training” periods, and their sounds were randomly (and arbitrarily) assigned to “trained” and “novel” for testing. The training and testing paradigms were the same, which consisted of a two-interval, two-alternative forced-choice (2i-2afc) task. Examples of the different trials can be seen in Fig 1.

For each trial, the pre-change scene was presented (comprised of four sounds played simultaneously), and then the post-change scene (with 400 ms ISIs of silence after every scene), alternating back and forth until either the participant responded with a button press on the keyboard (placed directly in front of them), or until the trial ended after eight scene-pair repetitions. Trials were either the same or contained a random switch in the location of two of the objects in the post-change scene. Participants were instructed prior to each trial on the computer screen with the directions of which key to press for a “change” or “same” trial. A fixation cross was presented in the center of the computer screen during each trial and remained until the participant responded. There was a 50% *a priori* probability that each trial would contain a change. Trials were pseudo-randomized so that no trial type was repeated more than four times in a row.

#### 2.4.1 Training

Trained and control participants were brought in for Day 1 and completed two ~30 minute sessions (“Session 1” and “Session 2”), spaced 30 minutes apart. Trained participants completed six blocks of 24 trials (144 total trials) with their four “trained” sounds in both sessions. Visual feedback of “Correct,” “Wrong,” or “Failed to make response on time” were shown on the screen following a response or time out. Control participants performed a visual task (a one-shot change blindness task using complex environmental scenes) during the two sessions. This was a task in which photographs of visual scenes were presented, of which one object could change between two intervals on each trial. The data from the training for control participants was used as pilot data for another study and will not be discussed further here.

#### 2.4.2 Testing

On Day 2, all participants completed the same testing. There was only one testing session, and EEG was recorded from all participants. Testing consisted of ten blocks of 36 trials (360 total trials), with “trained” and “novel” sound trials intermixed within blocks. Trained participants heard trials with the four sounds they heard during training, and trials with four sounds that were not present during training. Control participants performed the task for the first time. There was no feedback during testing.

#### 2.4.3 Electrophysiology

EEG data were collected using 70 channels (64 scalp and six electrodes lateral to and beneath the eyes, and on each mastoid) at a 2048-Hz sampling rate, 24-bit A/D resolution, referenced to the common-mode-sense driven-right-leg using CMS/DRL of the BioSemi Active II system (BioSemi, Amsterdam, the Netherlands). The 64 electrode BioSemi caps had several standard international 10–20 system electrode locations, and were fit to each participant’s head measurements accordingly.

Data were re-sampled at 256 Hz offline and re-referenced to the mastoid average, then digitally bandpass filtered (0.25–50.25 Hz half amplitude cutoff, 1691 point zero-phase Hamming-windowed sinc FIR, 0.5 Hz transition band, 0.0025 dB passband ripple, -40 dB stopband attenuation) using EEGLAB [45]. Noisy continuous data and channels were manually removed from the data by visual inspection, as suggested by Delorme et al. [46]. The mean number of channels maintained in the data after manual removal was 67.15 (*SD* = 1.23, range 63–68). Rejection of data was based solely on continuous data. Remaining data were subjected to an independent component analysis (ICA), after which independent components (ICs) were removed based on scalp projection, spectra, and time course (see [47] for a review) to correct for clear eye-movement and muscle-related artifacts. No data was rejected following ICA removal. The mean number of ICs remaining after removal was 61.77 (*SD* = 2.00, range 56–66).

Epochs were extracted from -0.2 to 1.0 s from each scene presentation onset before the response. Baselines were subtracted from each data epoch (-0.2 s to stimulus onset). Epoch time course voltages were then averaged to construct ERPs. P1, N1, P2, and P3b component amplitudes were extracted from the ERPs using mean voltages within specified time windows selected based on grand average ERP waveforms (averaged across all participants and conditions). The P1 time window fell between 50–80 ms after stimulus onset, N1 between 90–120 ms, P2 between 140–220 ms, and P3b between 400–700 ms after stimulus onset. Component amplitudes were taken as mean voltage across time points in the selected time windows.

ERPs were averaged from the following electrode clusters: frontal (Fz, AFz, F2, FCz, and F1), central (Cz, FCz, C2, CPz, and C1), posterior (Pz, CPz, P2, POz, and P1), left (FT7, FC5, T7, C5, TP7, and CP5), and right (FT8, FC6, C6, T8, CP6, TP8). Headplots for the locations of the clusters can be seen in Fig 4. The clustering of electrodes method was taken so as to mitigate potential signal-to-noise ratio problems that could arise if some subjects had poor electrode connectivity at one or more locations. Also, note that though spatial certainty on the scalp is diminished with this clustering, the ERP components we expected to analyze were expected to be apparent in this clustering. Here we were primarily interested in using EEG for its exceptional temporal resolution. Source estimation was not planned.

Statistical analyses entailed repeated-measures and mixed model ANOVAs interpreted with Greenhouse-Geisser corrections to degrees of freedom (uncorrected degrees of freedom reported). In cases of significance, false discovery rate corrected post-hoc *t-*tests were conducted where corrected *p*-values are reported. For readers interested in single-subject ERP data rather than aggregate data, a table is provided in S1 Table.

## 3. Results

### 3.1 Performance improvements during training

**Fig 2:**
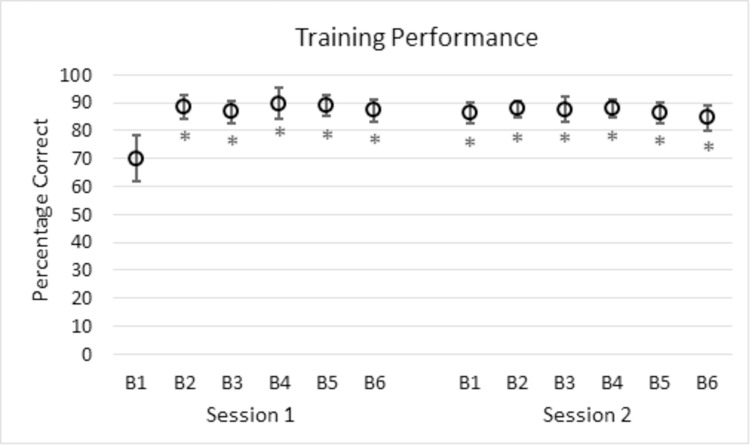
Training performance over Session 1 and Session 2 for trained participants. “B” indicates block number. Errors bars are standard error (SE, [49]). Asterisks indicate blocks that are significantly different from Block 1 with FDR adjusted p-values: *p < .05.

Fig 2 shows change detection performance across all training blocks for the trained group of participants. A repeated-measures ANOVA over all twelve blocks showed that significant differences existed among blocks, *F*(11, 99) = 4.60, *p* < .001, η_p_^2^ = .34. This is likely related to an improvement in performance for all blocks following Block 1 of Session 1. Post-hoc *t*-tests, comparing each block to the first block (Block 1 of Session 1) showed that all blocks had significantly higher performance than the first block (*p* < .05; FDR corrected, [48]). Overall, it appears that learning occurred, and that it occurred early on in training.

### 3.2 Trained participants had higher sensitivity and lower bias than controls during testing

**Fig 3:**
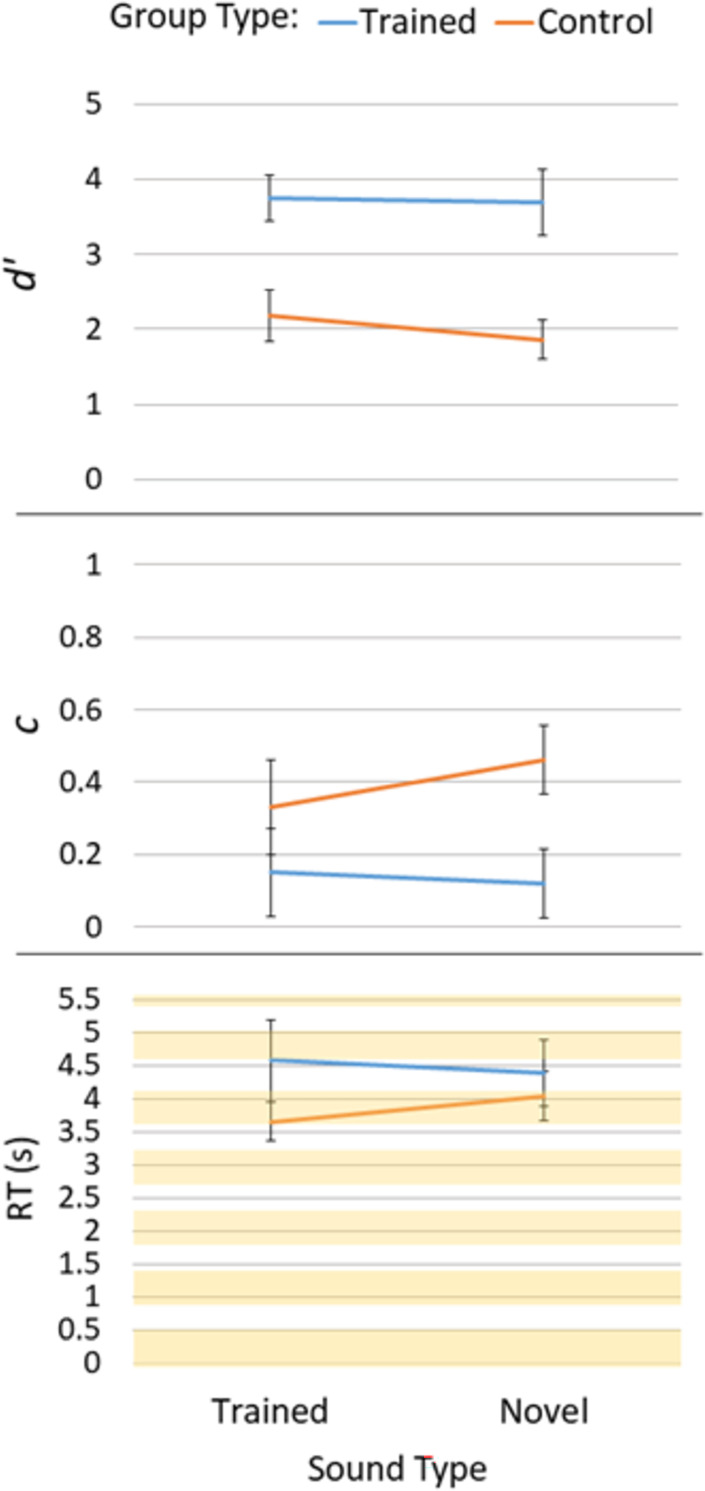
Top panel shows *d’*, middle panel shows c, and bottom panel shows RT in seconds for correct trials. Line colors indicate Group Type and left and right sides indicate Sound Type. Error bars are SE. In the RT panel (bottom), the scene presentation periods are represented by color blocks (first scene presentation starts at zero for 500 ms, and 400 ms occurs between each scene presentation).

To determine whether there were any training-related impacts to either sensitivity or bias the detect changes, the signal detection measures *d’* and *c* were analyzed, respectively. This data is shown in Fig 3. We used 2 (Group: Trained or Control) x 2 (Sound Type: “Trained” or “Novel”) mixed model ANOVAs to analyze this data.

For *d’*, there was a main effect of Group, *F*(1, 18) = 15.20, *p* = .001, η_p_^2^ = .46. Trained participants had higher sensitivity (*d’* = 3.61; *SD* = 1.06) than control participants (*d’* = 1.95; SD = .89). Both the main effect of Sound Type, *F*(1, 18) = .91, *p* = .35, η_p_^2^ = .15, and the Sound Type x Group interaction, *F*(1, 18) = .42, *p* = .53, η_p_^2^ = .02, were not significant.

For *c*, there was a main effect of Group, *F*(1, 18) = 4.47, *p* = .049, η_p_^2^ = .20. Bias was larger for controls (*c* = .40, *SD* = .24) than trained (*c* = .17, *SD* = .31) participants. The direction of this bias indicated a preference for the "same" response (cf. [25]). There was no significant main effect of Sound Type, *F*(1, 18) = .19, *p* = .67, η_p_^2^ = .31, or Sound Type x Group interaction, *F*(1, 18) = .63, *p* = .44, η_p_^2^ = .03.

Because this is the first study using a flicker task to study impacts of training on change deafness, we were also interested in how training may have affected the speed at which change detections were made. Median RTs for correct trials were determined and another 2 (Group) x 2 (Sound Type) repeated measures ANOVA was conducted. There was no significant main effect of Group, *F*(1, 18) = .98, *p* = .34, η_p_^2^ = .05, main effect of Sound Type, *F*(1, 18) = .35, *p* = .56, η_p_^2^ = .02, or an interaction, *F*(1, 18) = 2.93, *p* = .10, η_p_^2^ = .14 (see Fig 3).

Overall, the behavioral data demonstrate clear impacts of training on performance in the flicker task. Sensitivity to change was greater for the trained compared to control participants. Further, training effects applied to both sounds experienced during training and novel sounds. This was also true for response biases in that trained individuals displayed less bias than controls for all sounds tested.

### 3.3 ERPs distinguish “same” from “change” trials and the trained from control group

High overall accuracy across subjects (*M* = 86.35%, *SD* = 11.38%), led to a low number of error trials that could potentially be analyzed (some participants had as few as five wrong trials). Consequently, only correct trials were included in the ERP analysis.

**Fig 4:**
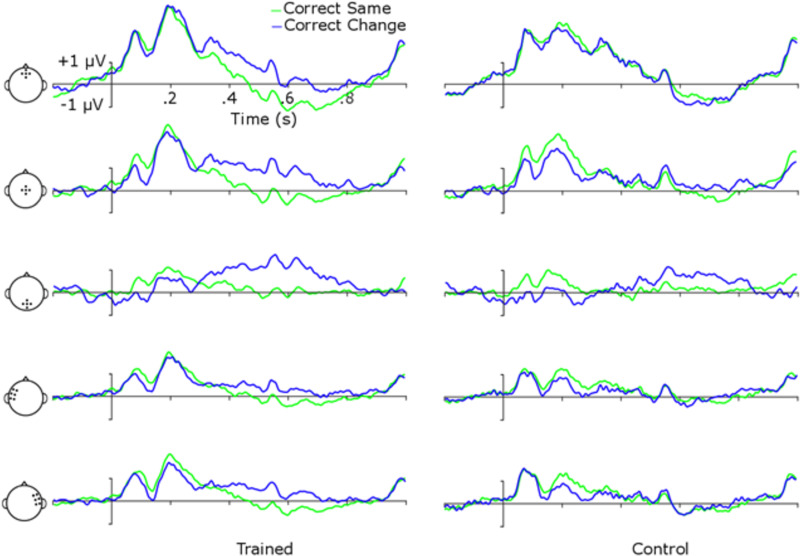
ERPs for different trial types and training conditions. Head indicates electrode clusters for the row, columns are groups, and colored lines indicate type of trial.

We first address ERPs generated using all iterations of scene presentations prior to the response. This allows the closest comparison of ERP effects in the current flicker paradigm to those reported in the previous one-shot paradigms, and it maximizes the number of epochs for analysis of potential training condition vs. control condition ERPs. Fig 4 shows ERPs at frontal, central, posterior, left, and right clusters of electrodes (see headplots in Fig 4 for cluster electrode locations). The first apparent trend in the data is a high-amplitude, low-frequency entrainment at the rate of scene presentation (1.1 Hz) upon which faster components of the ERP are superimposed. Nevertheless, ERPs show clear P1, N1, and P2 components of the auditory event-related potential, and baseline data among conditions appear to be similar, suggesting that this entrainment did not differentially impact conditions.

To examine potential differences in ERP component amplitudes, a 5 (Electrode Cluster) x 2 (Group) x 2 (Trial Type: “Same” or “Change”) mixed-model ANOVA was performed independently for P1, N1, P2, and P3b amplitudes. Subsequent post-hoc *p*-values were interpreted using the FDR approach.

For the P1 component there was a main effect of Electrode Cluster, *F*(4, 72) = 42.78, *p* < .001, η_p_^2^ = .70, reflecting an expected difference in amplitude among electrode clusters for auditory ERP components [50]. There was also a significant main effect of Trial Type, *F*(1, 18) = 6.82, *p* = .02, η_p_^2^ = .28, and an Electrode Cluster x Trial Type interaction, *F*(4, 72) = 18.72, *p* < .001, η_p_^2^ = .51. Post-hoc comparisons revealed that “same” trials were significantly more positive than “change” trials at central electrodes, *t*(19) = 3.68, *p* = .005, Cohen’s *d* = 0.84, and parietal electrodes, *t*(19) = 5.77, *p* < .001, Cohen’s *d* = 1.28. There was not a significant Electrode Cluster x Group interaction, *F*(4, 72) = .27, *p* = .89, η_p_^2^ = .02, Trial Type x Group interaction, *F*(1, 18) = .28, *p* = .60, η_p_^2^ = .02, Electrode Cluster x Trial Type x Group interaction, *F*(4, 72) = 1.58, *p* = .19, η_p_^2^ = .08, or a main effect of Group, *F*(1, 18) = .27, *p* = .61, η_p_^2^ = .02. See Table 2 for “same” and “change” trial amplitudes.

**Table 2:** *Mean amplitudes in microvolts (μV; with* SD *in parentheses) of ERP components for the Trial Types for* each Group. P1, N1, and P2 were averaged from the central electrode cluster, and P3b from the parietal electrode cluster.

		Group	
Component	Trial Type	*Control*	*Trained*
P1 P1	Same	1.83 (1.29)	1.27 (1.93)
	Change	1.33 (1.40)	.93 (1.75)
N1 N1	Same	1.77 (1.42)	1.55 (2.52)
	Change	.96 (1.83)	.61 (2.58)
P2 PP2a2	Same	2.71 (1.78)	2.84 (3.23)
	Change	1.98 (1.87)	2.57 (3.05)
P3b PP2a2	Same	.11 (1.76)	.47 (1.34)
	Change	.16 (1.30)	1.05 (1.95)

The analyses of N1 revealed a significant main effect of Electrode Cluster, *F*(4, 72) = 31.25, *p* < .001, η_p_^2^ = .63, main effect of Trial Type, *F*(1, 18) = 7.65, *p* = .01, η_p_^2^ = .30, and a Electrode Cluster x Trial Type interaction, *F*(4, 72) = 6.97, *p* = .005, η_p_^2^ = .28. Similar to the P1 analysis, post-hoc comparisons showed that “same” trials were significantly more positive than “change” trials at left electrodes, *t*(19) = 2.63, *p* = .03, Cohen’s *d* = 0.58, central electrodes, *t*(19) = 3.39, *p* = .02, Cohen’s *d* = 0.76, and parietal electrodes, *t*(19) = 3.21, *p* = .01, Cohen’s *d* = .72. There was no significant Electrode Cluster x Group interaction, *F*(4, 72) = .40, *p* = .81, η_p_^2^ = .02, Trial Type x Group interaction, *F*(1, 18) = .35, *p* = .56, η_p_^2^ = .02, Electrode Cluster x Trial Type x Group interaction, *F*(4, 72) = .10, *p* = .98, η_p_^2^ = .01, or a main effect of Group, *F*(1, 18) = .07, *p* = .80, η_p_^2^ = .004.

For P2, again there was a main effect of Electrode Cluster, *F*(4, 72) = 26.91, *p* < .001, η_p_^2^ = .60, a main effect of Trial Type, *F*(1, 18) = 9.03, *p* = .008, η_p_^2^ = .33, and a significant Electrode Cluster x Trial Type interaction, *F*(4, 72) = 4.87, *p* = .02, η_p_^2^ = .21. Post-hoc comparisons showed that “same” trials were significantly larger than “change” trials at left electrodes, *t*(19) = 2.63, *p* = .02, Cohen’s *d* = 0.59, right electrodes, *t*(19) = 3.44, *p* = .01, Cohen’s *d* = 0.79, central electrodes, *t*(19) = 2.66, *p* = .03, Cohen’s *d* = 0.57, and parietal electrodes, *t*(19) = 3.19, *p* = .01, Cohen’s *d* = .70. There was no Electrode Cluster x Group interaction, *F*(4, 72) = .08, *p* = .99, η_p_^2^ = .004, Trial Type x Group interaction, *F*(1, 18) = 1.19, *p* = .29, η_p_^2^ = .06, Electrode Cluster x Trial Type x Group interaction, *F*(4, 72) = .75, *p* = .56, η_p_^2^ = .04, or main effect of Group, *F*(1, 18) = .34, *p* = .57, η_p_^2^ = .02.

P3b showed a significant main effect of Electrode Cluster, *F*(4, 72) = 4.91, *p* = .008, η_p_^2^ = .21, a significant main effect of Trial Type, *F*(1, 18) = 20.70, *p* < .001, η_p_^2^ = .54, a Trial Type by Group interaction, *F*(1, 18) = 11.82, *p* = .003, η_p_^2^ = .40, and an Electrode x Trial Type interaction, *F*(4, 72) = 5.04, *p* = .02, η_p_^2^ = .22. Post-hoc comparisons showed that the trained group had higher “change” trial amplitudes than “same” trial amplitudes for P3b, *t*(19) = 4.20, *p* = .004, Cohen’s *d* = 1.32, but control group amplitudes did not differ between Trial Types, *p* = .11. Differences in P3b amplitude between Trial Types were significant at right electrodes, *t*(19) = 2.91, *p* = .02, Cohen’s *d* = 0.51, central electrodes, *t*(19) = 4.65, *p* < .001, Cohen’s *d* = 1.02, and parietal electrodes, *t*(19) = 5.29, *p* < .001, Cohen’s *d* = 1.18. There was no significant Electrode Cluster x Group interaction, *F*(4, 72) = .34, *p* = .85, η_p_^2^ = .02, Electrode Cluster x Trial Type x Group interaction, *F*(4, 72) = 1.40, *p* = .24, η_p_^2^ = .07, or main effect of Group, *F*(1, 18) = .05, *p* = .83, η_p_^2^ = .003.

The analysis of ERPs yielded some notable similarities and differences when compared with ERPs from the one-shot change deafness paradigm. As with previous work with the one-shot paradigm, P3b amplitude mapped onto change such that “change” trials elicited a larger amplitude than “same” trials [37]. The P1, N1, and P2 effects all showed more negative-going mean amplitudes for “change” than “same” trials. Rather than an effect attributable to these components being larger in amplitude for “change” trials, it thus seems that there was a more sustained negativity that might be underlying these effects as observed when analyzing the components independently. This could, for instance, be a mismatch negativity-like component (MMN) or a processing negativity (PN) that shows up with repeating scenes. The only evidence for an ERP change paralleling the behavioral improvements demonstrated by the trained group were larger P3b amplitudes for “change” trials. Also of note is that we also examined this data with a 0.1 Hz half-amplitude cutoff highpass filter, and there were no apparent differences in the latency or general waveform amplitudes of any ERP components. This suggests that the observed effects are not related to any filtering artifact.

### 3.4 Analysis of build-up effects: ERPs prior to response

**Fig 5:**
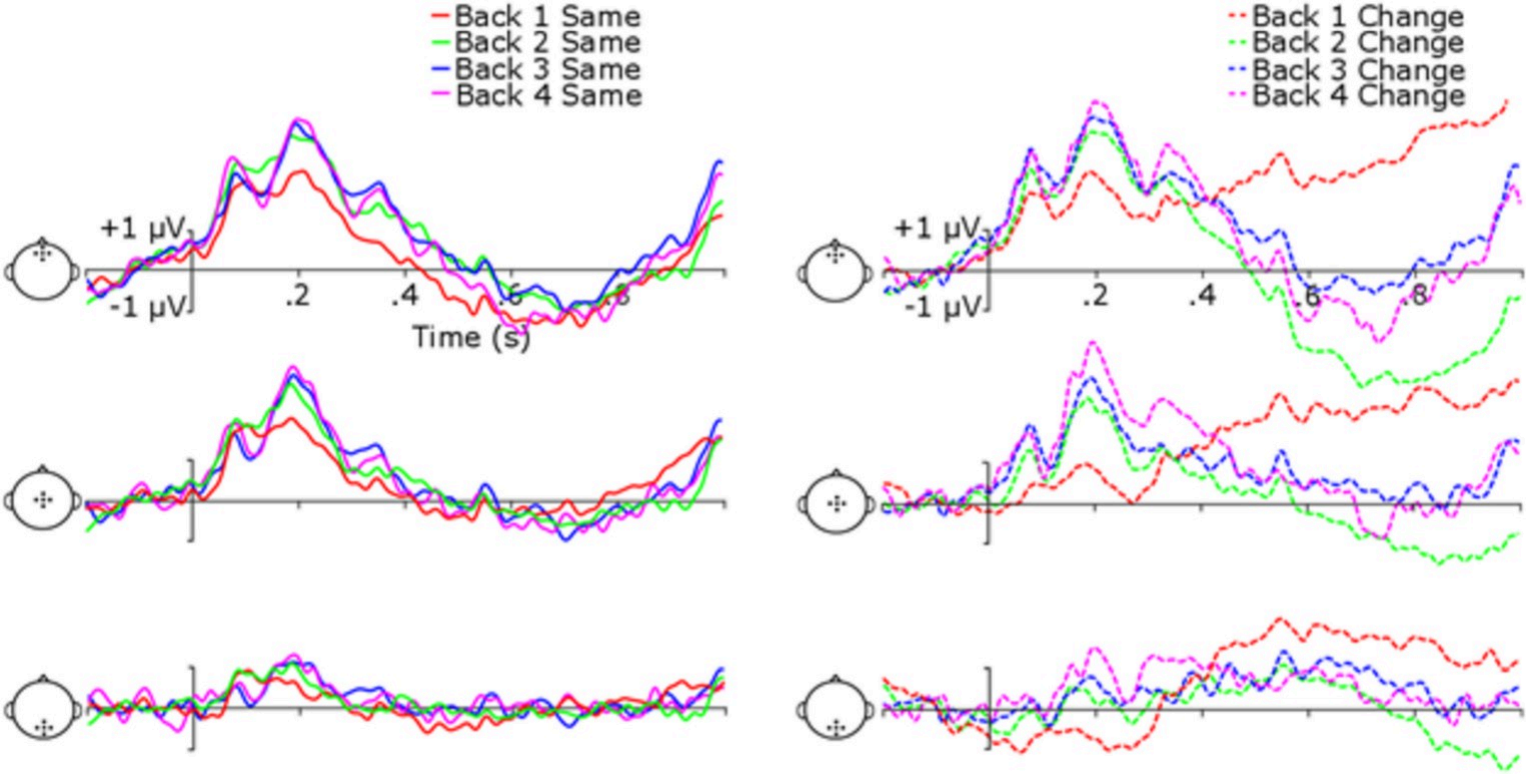
ERPs for mean amplitudes of trial types by number of repetitions back from response. Top ERP at frontal clusters, middle at central clusters, bottom at parietal clusters. Right column are same trials, left trials are change trials.

Fig 5 shows ERPs broken up by the number of scene presentations back in time from a response. Note that the number of scene presentations could reach up to 16, but that participants rarely listened to all 16 scene presentations before responding (also see RTs in Fig 3). The number of epochs included in the ERP and scene presentation back from the response were negatively related: while all trials had at least two iterations (pre- and post-change scene), the greater the number of presentations, the fewer epochs there were for that particular number of presentation back from the response. Hence, we included ERPs to scene presentations only up to four back from the response. This ensured that enough trials were present to form ERPs. P1, N1, and P2 amplitudes were averaged from the central electrode cluster, and P3b from the parietal cluster. Mean amplitudes for each component were analyzed independently with 2 (Trial Type) x 4 (Presentations Back) repeated-measures ANOVAs. No main effects or interactions were found when Group was included as a factor.

For P1, there was a main effect of Number of Repetitions from Response, *F*(3, 57) = 4.45, *p* = .03, η_p_^2^ = .19. Post-hoc t-tests on the consecutive pairs of Repetitions from the Response show that P1 amplitudes decreased significantly between Back 2 and Back 1 (right before the response), *t*(19) = 3.86, *p* = .003, Cohen’s *d* = 0.90. This was the only difference in P1 amplitudes among the levels back from response. There was no main effect of Trial Type, *F*(3, 57) = 4.09, *p* = .06, η_p_^2^ = .18, and no Number of Repetitions from Response by Trial Type interaction, *F*(3, 57) = 2.47, *p* = .07, η_p_^2^ = .12.

For N1, there was no significant main effect of Number of Repetitions from Response, *F*(3, 57) = .20, *p* = .90, η_p_^2^ = .01, but there was a significant main effect of Trial Type *F*(1, 19) = 8.65, *p* = .008, η_p_^2^ = .31, and a significant interaction, *F*(3, 57) = 2.79, *p* = .049, η_p_^2^ = .13. “Same” trial amplitude was larger than “change” trial amplitude for Back 1, *t*(19) = 3.35, *p* = .02, Cohen’s *d* = 0.75, and Back 2, *t*(19) = 2.86, *p* = .04, Cohen’s *d* = 0.64.

For P2 there was a main effect of Number of Repetitions from Response, *F*(3, 57) = 14.79, *p* < .001, η_p_^2^ = .44, and a significant interaction, *F*(3, 57) = 2.95, *p* = .04, η_p_^2^ = .13. P2 amplitudes decreased significantly between Back 2 and Back 1, *t*(19) = 3.56, *p* = .006, Cohen’s *d* = 0.81. P2 “same” trial amplitude was similarly larger than “change” trial amplitude for Back 1, *t*(19) = 2.77, *p* = .03, Cohen’s *d* = 0.62. There was no main effect of Trial Type, *F*(1,18) = 1.18, *p* = .29, η_p_^2^ = .06,

For P3b, there was no significant main effect of Number of Repetitions from Response, *F*(3, 57) = .44, *p* = .73, η_p_^2^ = .02, but there was a main effect of Trial Type *F*(1, 19) = 31.24, *p* < .001, η_p_^2^ = .62, replicating the initial analysis with all presentations reported above. There was no Trial Type x Presentations Back interaction, *F*(3, 57) = 2.10, *p* = .11, η_p_^2^ = .10.

**Fig 6:**
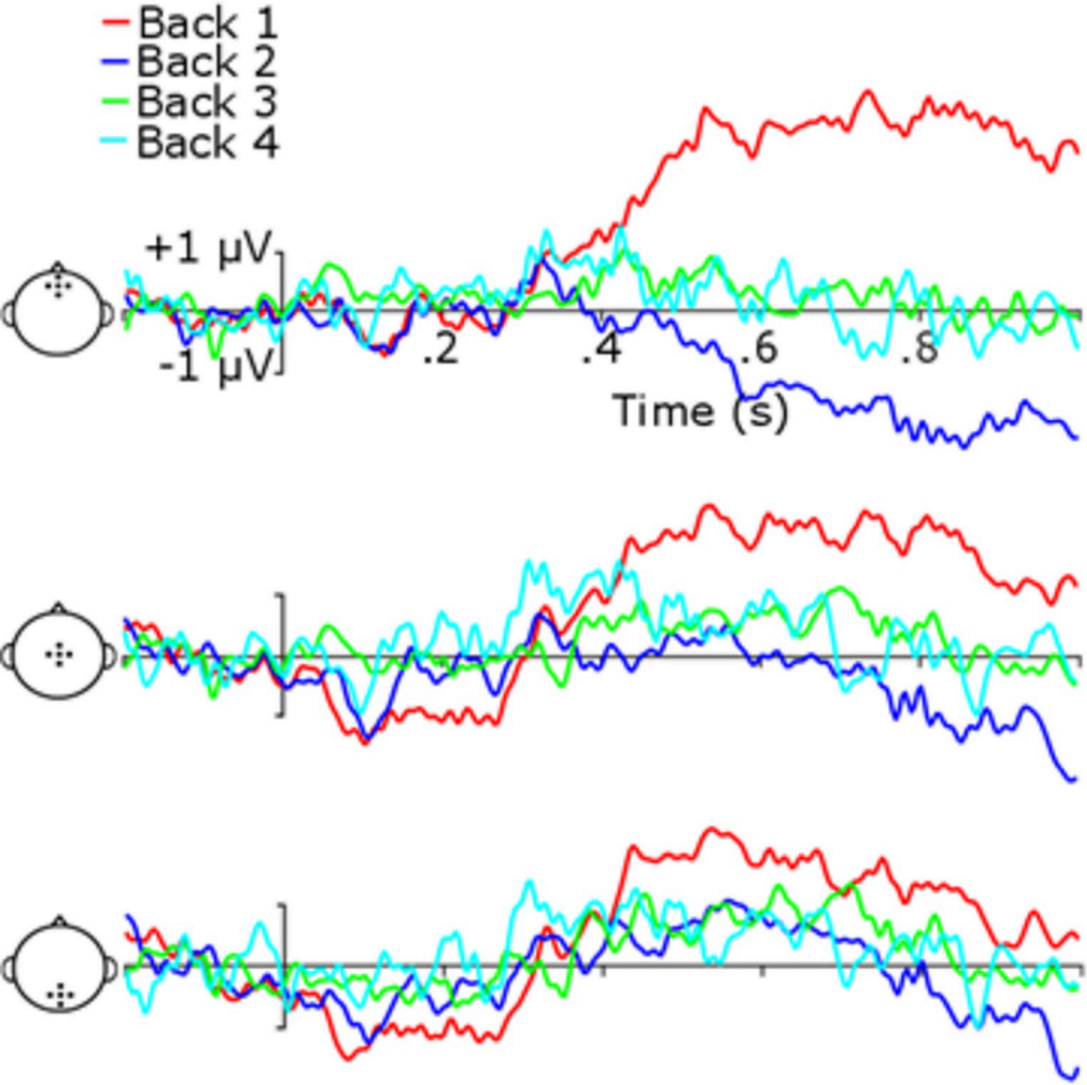
ERPs for difference wave amplitudes for trial types by number of repetitions back from response. Top ERP at frontal clusters, middle at central clusters, bottom at parietal clusters.

It appeared from the analysis of ERPs to scene presentations prior to the response that there was some build-up in the difference between "change" and "same" trials in the N1 and P2 components of the ERP. From Fig 5, it also appeared that there may have been a larger P3b just prior to the response for "change" trials, but this did not reach significance. To visualize the build-up of the difference between trial types, we calculated difference waves (“change” minus “same”) for the number of presentations back from a response (Back 1 through Back 4), see Fig 6. These difference waves show a building negative-going amplitude starting at around 100 ms, and reaching almost up to 300 ms for the Back 1 difference wave. A positive going and longer lasting difference appears later.

Repeated-measures ANOVAs were conducted on the difference waves for assessing potential differences among number of repetitions from Response (1–4) for N1, P2, and P3b. Difference wave amplitudes were analyzed in the windows of N1 and P2 at the central electrode cluster. The P3b difference was analyzed at the parietal cluster. The ANOVA for N1 was significant, *F*(3, 57) = 2.79, *p* = .049, η_p_^2^ = .13, as was the ANOVA for P2, *F*(3, 57) = 2.94, *p* = .04, η_p_^2^ = .13. The later positivity in the P3b range did not reach significance *F*(3, 57) = 2.10, *p* = .11, η_p_^2^ = .10. This was somewhat surprising given the appearance of large differences in positivity within the P3b range for difference waves at all clusters for back 1 compared to the other presentations. However, the lack of significance could be related to low trial counts for ERP formation in the build-up analysis. This trial count problem could also potentially explain why the visually apparent effect seems strongest at frontal electrodes. This could occur if noise invaded these later time points to skew the mean ERP in a manner that is not consistent with typical P3b scalp topography. Initial uncorrected post-hoc paired *t*-tests for consecutive pairs showed that for N1, Back 2 differed from Back 3, and for P2, Back 1 differed from Back 2, but these were rendered non-significant with FDR correction (*t*(19) = 2.31, *p* = .10, Cohen’s *d* = 0.52; *t*(19) = 2.21, *p* = .12, Cohen’s *d* = 0.49, respectively).

## 4. Discussion

We had several goals in mind at the outset of this study. The first was to evaluate how training to detect changes in auditory scenes impacted change detection performance and whether or not these effects were stimulus-specific. Second, we were interested in the ERP signatures of auditory learning and at what stages of processing they arose. Finally, we were interested in characterizing the ERP signatures of change detection and how these build up over multiple scene presentations in a flicker task. These goals are all novel in the context of change deafness research. Little is known about how change deafness is affected by training, except that training can generally improve performance [25]. This is also the first experiment known to the authors where an auditory flicker task was used in an ERP study of change deafness.

Listeners trained to detect spatial changes in auditory scenes made up of four distinct sound sources were better able to detect these changes after training than listeners that were trained in a visual task. That is, training improved change detection performance as expected (cf. [25]). Interestingly, this improvement was not only apparent for scenes made up of sound sources these listeners were trained with, but also for scenes made up of novel sound sources. There was no indication of any difference between performance with “trained” versus “novel” sound sources. This complete generalization was somewhat unexpected as auditory perceptual learning studies have shown stimulus specific training effects in detection of tones in noise [22], pitch discrimination among simple and complex sounds [51–53], relative and absolute timing [54,55], and amplitude and frequency modulation rates [56,57]. Also, compared to literature on change blindness, our findings differ from Rosen et al. [29] which found stimuli-specific improvements from training on a visual change detection task. The results also run counter to the assertion made by Gaspar et al. [28] that change blindness improvements were a result of familiarity with the training stimuli.

There are several possible explanations for why we observed complete generalization of learning to a novel set of sounds. One is that learning impacts on change deafness are exceptionally generalizable. Gregg and Samuel [16] found that familiarity with sounds did not affect performance in a change deafness task and concluded that perhaps experience with specific sounds is not key to the ability to detect changes between scenes in audition. However, we believe this possibility to be unlikely. Advantages for learned sounds in auditory scene processing have been well demonstrated, with learned sounds being more easily streamed out of a background [58,59], learned sounds more easily detected within scenes [60], and changes in auditory scenes between learned object categories being more easily detected than changes within object categories [61].

Another possible explanation for why we observed complete generalization from “trained” to “novel” sound sources is because of the spatial nature of our task. Because we used HRTFs to render our sound sources at the desired azimuths, the cues to spatial changes in scenes included interaural level differences (ILDs), interaural timing differences (ITD), and spectral features. Listeners’ improvements may have been related to learning to attend to these particular cues, with the same dimensions being as useful for monitoring “trained” sounds as they were for novel ones. If this is the case, specificity might be seen in a similar task that introduces changes in object identity rather than spatial location. Future research will be necessary to determine if this is the case.

Another interesting impact of training was that decision criteria became less biased. Specifically, trained listeners, though still being "same" biased, were less biased than the control group. Jones et al. [62] similarly found that 83% of listeners that showed decreased thresholds for amplitude modulation detection over the course of training also showed a reduction in bias. Bias reduction may be correlated strongly with improvements in detection thresholds (also, see [63]). In an examination of individual differences in discrimination learning along a continuum of temporally altered birdsong stimuli, different groups of learners were identified that had either strong changes in discriminability (*d’*) or strong changes in bias (*c*) during training [53]. The results suggested that these different types of learning could yield similar effects on stimulus generalization gradients along the trained dimension. Together with our finding and Irsik’s [25] finding of a learning-related reduction in bias in change deafness tasks, this work suggests that response bias should be consistently evaluated in perceptual learning work going forward. At a minimum, bias should be examined at least to parse it from changes in perceptual sensitivity that may be of more interest to researchers.

The only ERP component that appeared to correlate with impacts of training on change deafness was the P3b. The P3b had a larger amplitude on “change” trials for the trained compared to the control group. Though there were earlier ERP components associated with changes in auditory scenes (see below for further discussion), these did not appear to differ between groups. Perceptual learning has previously been associated with increases in the amplitude of the P3b in auditory [33,64], visual [65], and somatosensory [66] learning studies. The impact of training on P3b amplitude could reflect several things, based on the nature of the change detection task used. Increased P3b amplitude for trained participants on “change” trials could suggest training enhances the ability to compare pre- and post-change scenes in working memory [67], especially considering that P3b has been shown in previous change deafness tasks to be associated with *successful* detection of change between scenes. Another possibility is that learning yielded a larger degree of certainty in the decision-making process, resulting in a larger P3b (see [68,69]).

For correct “same” trials, P1, N1, and P2 amplitudes were significantly more positive than for "change" trials. The difference in earlier components in part parallels repeated findings that the auditory N1 is more negative for trials in which a change in scenes occurs [6,10,37]. However, that this same trend appeared for both P1 and P2, and that a more sustained negativity was observable in "change"/"same" difference waveforms, demonstrates some difference between this finding and previous work with the one-shot task. It is also interesting that this trend built up over repeated presentations such that it was strongest just before listeners made a response. Gutschalk et al. [41] examined responses to repeated tones at a fixed frequency and fixed ISI in a background mask of tones presented at random frequencies. In that study, a similar negativity showed a build-up in amplitude up until the listeners’ response. Those authors named the component an awareness-related negativity. It could also be that this negativity reflects something like the mismatch negativity (MMN) and/or a processing negativity (PN). MMN appears when a sound deviates from some established repetition of sounds [70–72]. On "change" trials in the flicker task there are back-and-forth changes between auditory scenes of equal probability. Though this may not form as strong an establishment of sound repetition as the typical oddball task used to elicit MMN, the paradigm does generate a deviance from the sound prior. PN can generally be a little later in latency than the N1 and has been presumed to reflect maintenance of memory for critical features of to-be-attended sounds. Future work using ERPs and the flicker paradigm will be needed to reveal more about the nature of our observed build-up of negativity prior to the response.

P3b amplitude was larger for correct “change” trials than for correct “same” trials. This is a replication of an effect observed several times in the one-shot task [6,10,37,38]. This is further evidence that the flicker task can be used to inform research on detection of changes in auditory scenes with ERPs. It is just as valid a task to use with the ERP method as the one-shot task. This is important because flicker tasks allow for neural processing comparisons of change detection or change deafness with the same stimulus change, and can be used to study the neural correlates of change awareness (like it has been in the visual literature, e.g. [26]). The flicker task also allows for sufficient encoding of stimuli with repeated presentations, and can reduce the effects that informational masking can have on whether or not a change is detected [8].

There a few caveats that we wish to acknowledge. The first is that although our sample size is comparable to several other works using the ERP approach to examine impacts of auditory training (e.g., [33–35]), we may have lacked sufficient power to detect smaller effects than that observed for the P3b in trained versus control group comparisons. Similarly, in our analysis of build-up effects, we were not able to look at scene presentations any further than four back from the response due to there being very few trials for those presentations within most subjects. To our knowledge this is the first ERP study to examine training effects on change deafness, and build-up effects in a flicker task. We now have an empirical basis for ideas of what kinds of ERP changes can occur as a result of training in change deafness tasks, and the ERP components that show build-up prior to responding. This will make it easier to optimize trial counts, sample sizes, and task parameters for future studies needed to replicate and extend this work. Prior to this, some tentativeness is warranted in regard to the current findings.

It is also worth consideration that change deafness rates were rather low, and improvements occurred rather quickly in training. Proportion correct at the end of training for the trained group was nearing 90% correct on average, and most of the learning appeared to occur between the first and second blocks. Because of this, we were slightly concerned that there would be rather quick learning for the control group during the testing phase that would muddy comparison with the trained group. However, an additional analysis (2 (Sound Type: Trained vs. Novel) x 10 (Blocks) ANOVA for the untrained subjects during testing) did not provide evidence supporting this concern. There was no significant main effect of Sound Type, *F*(1, 9) = .06, *p* = .81, η_p_2 = .007, Block, *F*(9, 81) = .57, *p* = .58, η_p_2 = .06, or interaction, *F*(9, 81) = .66, *p* = .59, η_p_2 = .07. Nevertheless, in future work it may be useful to vary task difficulty (e.g., in the spatial separation of sources) to get a clearer picture of learning effects. For instance, it is possible that training’s specificity is only apparent at near threshold same/change discrimination thresholds. Relatedly, given the short duration of this study (two days), we cannot determine how long training effects may last. Future work will be needed to determine how long-lasting perceptual learning induced reductions in change deafness last.

## 5. Conclusion

This study has expanded what is known regarding how training can impact deafness to changes in auditory scenes. First, it demonstrates that the impacts of training need not be stimulus specific. At least when changes to scenes occur spatially, training can have beneficial impacts that generalize beyond the sound sources used in training. Second, we have obtained evidence from ERPs that the impacts of training are correlated with an increase in P3b amplitude. This puts an upper bound for the level at which training has an impact, and suggests that training is at least having an impact on the ease to which a change can be explicitly detected.

This work also provides the first look at ERP signatures of change detection in an auditory flicker task with scenes. Though there are unique issues to consider when using the flicker task (e.g., entrainment to the rate of scene presentation), ERP signatures of change detection found with previous one-shot tasks were also apparent here (e.g., greater P3b amplitude for “change” over “same” trials). Further, ERP amplitudes differed as repeated scene presentations were experienced. Build-up effects were also apparent in an early negative going ERP component overlapping with the N1 and P2. Employing the EEG method in studies of change deafness using flicker tasks will reveal new insights into deafness of change in auditory scenes.

## Supporting information

S1 Table Individual mean amplitudes for the P1, N1, P2, and P3 components at left, right, frontal, central, and parietal electrode clusters. Different mean amplitudes for “same” and “change” trials are also shown. Subject numbers and training condition are given in the first two columns. Refer to the paper for further details. For more information, email the 1^st^ author (njball@buffalo.edu).
(XLSX)
